# Preclinical Evaluation of ^89^Zr-Desferrioxamine-Bexmarilimab, a Humanized Antibody Against Common Lymphatic Endothelial and Vascular Endothelial Receptor-1, in a Rabbit Model of Renal Fibrosis

**DOI:** 10.2967/jnumed.122.264725

**Published:** 2023-04

**Authors:** Olli Moisio, Jenni Virta, Emrah Yatkin, Heidi Liljenbäck, Senthil Palani, Riikka Viitanen, Maxwell W.G. Miner, Vesa Oikonen, Tuula Tolvanen, Danielle J. Vugts, Pekka Taimen, Xiang-Guo Li, Maija Hollmén, Sirpa Jalkanen, Anne Roivainen

**Affiliations:** 1 Turku PET Centre, University of Turku, Turku, Finland;; 2 Central Animal Laboratory, University of Turku, Turku, Finland;; 3 Turku Center for Disease Modeling, University of Turku, Turku, Finland;; 4 Turku PET Centre, Turku University Hospital, Turku, Finland;; 5 Department of Medical Physics, Turku University Hospital, Turku, Finland;; 6 Department of Radiology and Nuclear Medicine, Amsterdam UMC, VU University, Amsterdam, The Netherlands;; 7 Institute of Biomedicine, University of Turku, and Department of Pathology, Turku University Hospital, Turku, Finland;; 8 InFLAMES Research Flagship Center, University of Turku, Turku, Finland;; 9 Department of Chemistry, University of Turku, Turku, Finland; and; 10 MediCity Research Laboratory, University of Turku, Turku, Finland

**Keywords:** bexmarilimab, CLEVER-1, PET/CT, renal fibrosis, whole-body distribution, ^89^Zr

## Abstract

Bexmarilimab is a new humanized monoclonal antibody against common lymphatic endothelial and vascular endothelial receptor-1 (CLEVER-1) and is in clinical trials for macrophage-guided cancer immunotherapy. In addition being associated with cancer, CLEVER-1 is also associated with fibrosis. To facilitate prospective human PET studies, we preclinically evaluated ^89^Zr-labeled bexmarilimab in rabbits. **Methods:** Bexmarilimab was conjugated with desferrioxamine (DFO) and radiolabeled with ^89^Zr. Retained immunoreactivity was confirmed by flow cytometry. The distribution kinetics of intravenously administered ^89^Zr-DFO-bexmarilimab (0.1 mg/kg) were determined for up to 7 d in a rabbit model of renal fibrosis mediated by unilateral ureteric obstruction. The in vivo stability of ^89^Zr-DFO-bexmarilimab was evaluated by sodium dodecyl sulfate–polyacrylamide gel electrophoresis in combination with autoradiography. Additionally, we estimated the human radiation dose from data obtained in healthy rabbits. **Results:** ^89^Zr-DFO-bexmarilimab cleared rapidly from the blood circulation and distributed to the liver and spleen. At 24 h after injection, PET/CT, ex vivo γ-counting, and autoradiography demonstrated that there was significantly higher ^89^Zr-DFO-bexmarilimab uptake in unilateral ureteric obstruction–operated fibrotic renal cortex, characterized by abundant CLEVER-1–positive cells, than in contralateral or healthy kidneys. The estimated effective dose for a 70-kg human was 0.70 mSv/MBq. **Conclusion:** The characteristics of ^89^Zr-DFO-bexmarilimab support future human PET studies to, for example, stratify patients for bexmarilimab treatment, evaluate the efficacy of treatment, or monitor disease progression.

## Visual Abstract

**Figure:**
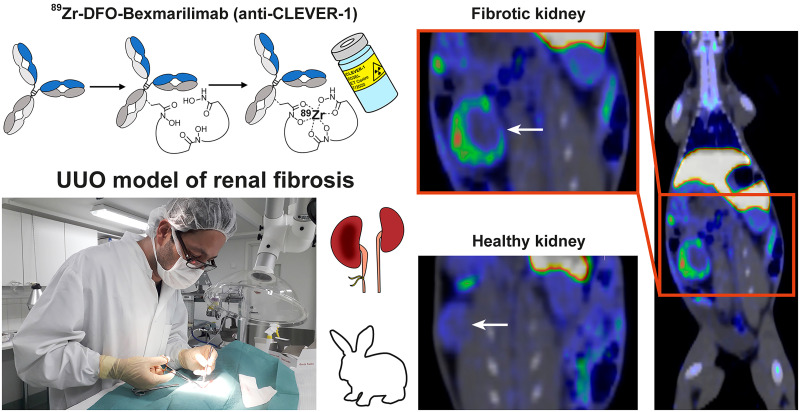

Common lymphatic endothelial and vascular endothelial receptor-1 (CLEVER-1, also known as stabilin-1 and FEEL-1[fasciclin, EGF-like, laminin type EGF-like, and link domain containing scavenger receptor 1]) is a multifunctional scavenger receptor expressed on antiinflammatory, alternatively activated M2 macrophages (1*,*^2^). In addition, the molecule is present, as the name suggests, in the lymphatic and vascular endothelium. In human tissues, CLEVER-1 is specifically expressed in the noncontinuous endothelial cells of the spleen, liver, adrenal cortex, and lymph nodes (3–5).

The humanized anti–CLEVER-1 antibody bexmarilimab (dissociation constant, 0.75 × 10^9^ M to human CLEVER-1) has been developed for immunotherapy and has recently shown promising results in clinical trials (6). Although the main research focus of studies investigating CLEVER-1 has been its effects on tumor-associated macrophages and cancer (7), CLEVER-1 mediates tissue homeostasis and prevents fibrosis in liver injury. In this context, CLEVER-1 protects against excessive fibrosis in response to oxidative stress by clearing modified low-density lipoproteins. The uptake of modified low-density lipoproteins reduces profibrogenic chemokine (C-C motif) ligand 3 secretion, resulting in reduced fibrosis and promotion of healing (8).

Therefore, we propose that CLEVER-1 may also be a relevant marker of tissue repair and the healing response in inflammatory diseases, and we present the preclinical evaluation of ^89^Zr-labeled desferrioxamine (DFO)-conjugated bexmarilimab in a rabbit model of renal fibrosis. Notably, the parental antihuman CLEVER-1 antibody 3-372 can also recognize rabbit CLEVER-1 (9). To obtain a detailed assessment of the whole-body distribution kinetics of intravenously administered bexmarilimab and its CLEVER-1–targeting ability, we radiolabeled bexmarilimab and preclinically evaluated the effects of ^89^Zr-DFO-bexmarilimab in healthy rabbits and rabbits with renal fibrosis induced by unilateral ureteric obstruction (UUO).

## MATERIALS AND METHODS

Supplemental materials and methods are available at http://jnm.snmjournals.org (10).

### Study Design

**FIGURE 1.**
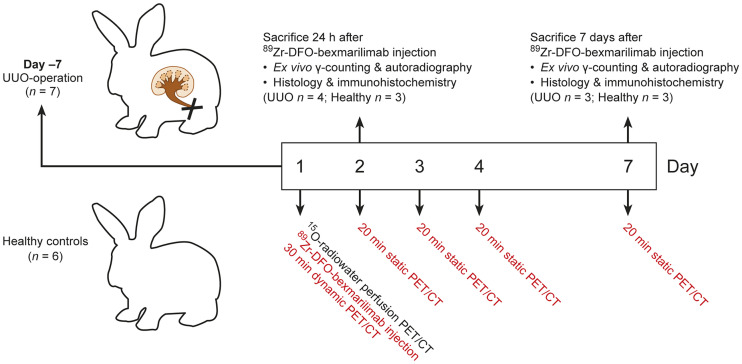
Study design for 6 healthy and 7 UUO New Zealand White rabbits. UUO surgery was performed 7 d before start of study, which began on day 1. Rabbits first were examined for renal perfusion using ^15^O-radiowater PET/CT. They then were intravenously injected with ^89^Zr-DFO-bexmarilimab for sequential PET/CT imaging for up to 7 d after injection and were killed for postmortem studies either 24 h or 7 d after injection.

The study protocol is presented in Figure 1. The UUO model of renal fibrosis in rabbits was used as the animal model. The left ureter was ligated in 7 female New Zealand White rabbits 7 d before ^89^Zr-DFO-bexmarilimab injection. Six healthy rabbits were studied as controls.

The whole-body distribution kinetics of intravenously administered ^89^Zr-DFO-bexmarilimab were studied in rabbits (*n =* 13; weight, 2.12 ± 0.19 kg) using in vivo PET/CT imaging for up to 7 d, as well as ex vivo γ-counting of excised tissues and digital autoradiography of kidney cryosections. Histologic and immunohistochemistry results supported the ex vivo autoradiographic results. Renal perfusion was determined by ^15^O-radiowater PET/CT before ^89^Zr-DFO-bexmarilimab injection, and the kidney volume was determined by CT. Human radiation dose estimates for ^89^Zr-DFO-bexmarilimab were extrapolated from healthy rabbit data.

All animal experiments were approved by the national project authorization board in Finland (license numbers ESAVI/856/04.10.07/2017 and ESAVI/5882/2020) and were performed in compliance with European Union directive 2010/EU/63 on the protection of animals used for scientific purposes.

### Preparation of ^89^Zr-DFO-Bexmarilimab

Bexmarilimab (IgG4, ∼150 kDa, in a 25 mg/mL stock solution containing 10 mM l-histidine/HCl [pH 6.0], 20 mM l-methionine, 280 mM trehalose, and 0.02% polysorbate 20) was obtained from Faron Pharmaceuticals (11). The DFO conjugation and radiolabeling of bexmarilimab with ^89^Zr were performed using a previously published protocol with slight modifications (12). To attach the hexadentate chelator DFO, bexmarilimab stock was reconstituted to a concentration of 3 mg/mL in sodium bicarbonate buffer (1.0 M, 1 mL, pH 9.0) by ultrafiltration (Amicon Ultra 60 kDa; Millipore). Subsequently, to 1 mL of the rebuffered antibody, isothiocyanatobenzyl-DFO (p-DFO-Bz-NCS, 10 μL, 3.5 mM in dimethyl sulfoxide, 2 equivalents) was added and the solution was incubated at 37°C for 30 min while being mixed. Then, the reaction mixture was transferred onto a PD-10 size-exclusion column (Cytiva) and DFO-bexmarilimab was eluted in 1.5 mL of formulation buffer containing 10 mM l-histidine, 20 mM methionine, 280 mM sucrose, and 0.02% polysorbate 20 in water at pH 6.0 (adjusted with HCl).

The radiolabeling was performed by mixing 40−45 MBq of ^89^Zr (100 μL, 1.0 M oxalic acid; Cyclotron VU), Na_2_CO_3_ (45 μL, 2.0 M), 4-(2-hydroxyethyl)-1-piperazineethanesulfonic acid buffer (500 μL, 0.5 M, pH 7.2), and DFO-bexmarilimab (355 μL, 1.3 mg/mL). The mixture was incubated at room temperature for 60 min while being mixed. The crude product mixture was transferred onto a PD-10 size-exclusion column, and ^89^Zr-DFO-bexmarilimab was eluted in 1.5 mL of formulation buffer.

The radiochemical quality of ^89^Zr-DFO-bexmarilimab was analyzed with 2 methods: ultrafiltration and sodium dodecyl sulfate–polyacrylamide gel electrophoresis (SDS-PAGE). For the ultrafiltration method, 5 μL of the product were added to a Microcon spin filter (60-kDa cutoff; Millipore) containing 95 μL of 5% dimethyl sulfoxide in phosphate-buffered saline. The product was centrifuged at 14,000*g* for 6 min followed by 2 washes with 100 μL of 5% dimethyl sulfoxide in phosphate-buffered saline. The radioactivity remaining in the filter and filtrate was then separately measured with a γ-counter (Wizard 1480; PerkinElmer). The radiochemical purity of ^89^Zr-DFO-bexmarilimab was determined as the amount of radioactivity on the filter divided by the total amount of radioactivity multiplied by 100%. The measurements were performed in triplicate. SDS-PAGE was performed to detect possible aggregates and larger fragments of antibodies. The assay was run on a Miniprotean electrophoresis system using precast 4%−20% nondenaturing Tris-glycine polyacrylamide gels (Bio-Rad). Subsequently, the gel was rinsed in water, placed on a phosphor imaging plate (Fujifilm), and after exposure scanned on a BAS-5000 scanner (Fujifilm). The resulting images were analyzed with an AIDA image analyzer (Raytest) to determine the percentage of intact ^89^Zr-DFO-bexmarilimab.

The immunoreactivity and CLEVER-1 binding of DFO-bexmarilimab and ^89^Zr-DFO-bexmarilimab after radioactive decay were confirmed by flow cytometry using unmodified bexmarilimab (clone CP-12; Abzena) as a reference molecule. Briefly, peripheral blood mononuclear cells (Finnish Red Cross) enriched with CD14-microbeads (Miltenyi) or the CLEVER-1^high^ acute myelogenous leukemia cell line KG-1 (CCL-246; ATCC) was used. KG-1 cells were cultured in Iscove modified Dulbecco medium supplemented with 20% fetal bovine serum and penicillin/streptomycin. The cells (0.5 × 10^6^/well) were plated in a round-bottom 96-well plate (Sarstedt) and stained with varying concentrations (μg/mL) of bexmarilimab, DFO-bexmarilimab, and ^89^Zr-DFO-bexmarilimab. An irrelevant isotype IgG4 control antibody, (S241/L248E)-Alexa Fluor 647 (Abzena), was used to normalize the signal. The cells were stained with mouse anti-human IgG4-Alexa Fluor 488 (catalog no. 9200-30; Southern Biotech). Fixed samples were subjected to flow cytometry on an LSRFortessa cell analyzer (Becton Dickinson) and analyzed with FlowJo10 (TreeStar Ashland) software.

### Radiosynthesis of ^15^O-Radiowater

^15^O-radiowater was synthesized as previously described (13).

### PET/CT Imaging

A Discovery-690 PET/CT (GE Healthcare) device was used for imaging studies.

Rabbits were sedated and anesthetized with a subcutaneous injection of a mixture of medetomidine (Cepetor Vet, 1 mg/mL, 0.1 mg/kg; CP-Pharma), ketamine (Ketaminol, 50 mg/kg, 2 mg/kg; Intervet Oy), and midazolam (5 mg/mL, 0.1 mg/kg; Hameln Pharma). For scanning on day 1, 2 cannulas were inserted, one in the left ear vein for injecting tracers and the other in the right ear vein or artery for blood sampling. For scans on day 2 onward, only one ear vein was cannulated for blood sampling.

For measurement of renal perfusion, 20 ± 0 MBq of ^15^O-radiowater were injected intravenously and the rabbits were imaged for 6 min (time frames: 15 × 4 s, 4 × 10 s, 4 × 20 s, and 3 × 60 s).

^89^Zr-DFO-bexmarilimab (7.2 ± 2.5 MBq [mean ± SD]; range, 4.4–10.2 MBq; 3.5 ± 1.3 MBq/kg; 0.1 mg/kg) was injected on the first scanning day, and dynamic whole-body PET/CT scanning was performed for 30 min immediately after the injection. Static whole-body scans were then obtained at 24 h, 2 d, 3 d, and 7 d after injection. Dynamic emission scans of 12 time frames (4 × 30 s, 4 × 60 s, 2 × 120 s, and 2 × 600 s) were acquired by serial imaging of the body in 2 contiguous segments. Three bed positions were required for static whole-body PET, with a 6-min acquisition time for each position.

PET/CT images were analyzed using Carimas software (www.turkupetcentre.fi/carimas/). Regions of interest were defined for the whole kidney (parenchyma), kidney cortex, kidney medulla, left ventricle cavity (representing blood), liver, lungs, muscle, myocardium, and spleen on the PET/CT images; the CT scan was used to provide an anatomic reference. The suprarenal abdominal aorta was used as the region of interest representing blood in ^15^O-radiowater analyses. Kidney volumes were determined from CT images. Results are expressed as SUVs and time–activity curves.

Renal perfusion was estimated by fitting a single-tissue compartmental model to the regional radioactivity concentration curves. An image-derived arterial blood curve was used as the model input function. The nonlinear sum-of-least-squares method was used to estimate perfusion (mL_blood_/mL_tissue_×min).

### In Vivo Stability of ^89^Zr-DFO-Bexmarilimab

Blood samples (100−1,500 μL) were collected into heparinized tubes at 1 min, 5 min, 10 min, 30 min, 1 h, 2 h, 3 h, 4 h, 24 h, 2 d, 3 d, and 7 d after ^89^Zr-DFO-bexmarilimab injection. The radioactivity of the whole-blood samples was measured with a Wizard γ-counter. Plasma was subsequently separated by centrifugation (2,100*g* for 5 min at 4°C), and radioactivity was measured using the γ-counter.

To determine the amount of intact ^89^Zr-DFO-bexmarilimab, aliquots of plasma were applied to native SDS-PAGE as described above. Briefly, a small volume of plasma (up to 10 μL) was added to 10 μL of 4× Laemmli sample buffer (Bio-Rad), filled to a volume of 40 μL with H_2_O, and applied on the gel. For high-radioactivity samples (0–4 h after injection), plasma was diluted with physiologic saline before preparation of the sample. ^89^Zr-DFO-bexmarilimab in formulation buffer (stored at 4°C) was used as a reference.

### Ex Vivo Biodistribution and Digital Autoradiography

Rabbits were euthanized either at 24 h (4 UUO, 3 healthy) or at 7 d (3 UUO, 3 healthy) after ^89^Zr-DFO-bexmarilimab injection. After the last PET/CT examination, the rabbits were euthanized under deep ketamine–medetomidine–midazolam anesthesia by cardiac puncture and an overdose of pentobarbital (Mebunat; Orion Pharma). Tissues and organs were immediately dissected, weighed, and assayed for radioactivity with a Wizard γ-counter. The radioactive decay of ^89^Zr (half-life, 3.3 d) was corrected for the time of injection. The uptake of radioactivity is expressed as SUV.

Samples of kidneys were embedded and frozen in Tissue-Tek (Sakura) and cut into 8-μm and 20-μm slices. The 20-μm cryosections were thaw-mounted onto microscope slides and immediately exposed to a phosphor imaging plate (Fujifilm). After an exposure time of approximately 3 d, the imaging plates were scanned with a BAS-5000 scanner. After scanning, sections were stained with hematoxylin–eosin and scanned with a digital slide scanner (Pannoramic 250 Flash; 3DHistec). The 8-μm sections were stored at −70°C and used for CLEVER-1 immunohistochemical staining.

The accumulation of ^89^Zr-DFO-bexmarilimab in the renal cortex and medulla was analyzed on superimposed autoradiographs and hematoxylin–eosin images using Carimas software. Results were decay-corrected for injection and exposure time, normalized to the injected radioactivity dose, and expressed as the photostimulated luminescence per square millimeter.

The 8-μm cryosections of the kidneys and spleen were stained with anti–CLEVER-1 (clone 3-372; InVivo Biotech) peroxidase. Briefly, the slides were acetone-fixed, blocked with horse serum, and incubated overnight at 4°C in a humidified chamber with a 10 μg/mL concentration of clone 3-372 or mouse IgG1 control antibody. The signal was detected using a Vectastain Elite ABC kit (Vector Laboratories) and liquid chromogen 3,3′-diaminobenzidine substrate (DAKO). The slides were counterstained with hematoxylin and imaged with the Pannoramic 250 Flash digital slide scanner. Endogenous peroxidase activity was not blocked before staining because this procedure reduces the staining quality.

Additional formalin-fixed, paraffin-embedded 7-μm kidney sections were stained with picrosirius red to evaluate kidney damage and the development of renal fibrosis.

### Statistical Analysis

Results are expressed as the mean ± SD. Differences between groups were determined with the independent-samples *t* test using Excel (Microsoft). *P* values of less than 0.05 were considered statistically significant.

## RESULTS

### Characterization of the UUO Rabbit Model

Visual and histologic inspection of the left kidney demonstrated obvious damage (swollen, enlarged kidney) due to blockage of urine flow to the bladder (Supplemental Fig. 1). The contralateral kidney appeared healthy, with no obvious damage.

**FIGURE 2.**
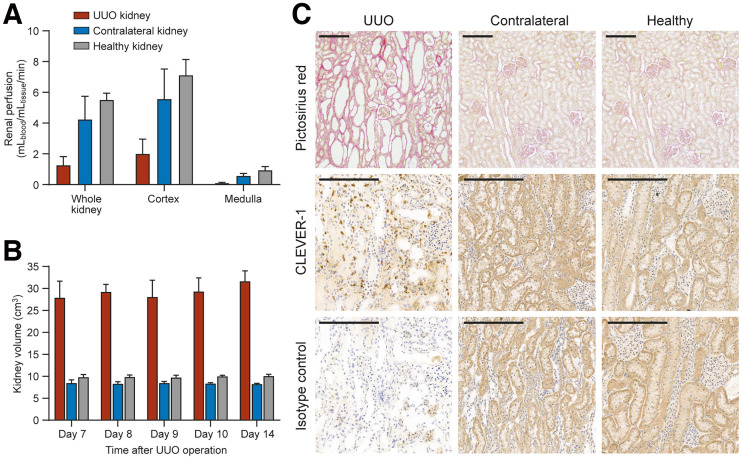
Characterization of rabbit UUO model. (A) Renal perfusion determined using ^15^O-radiowater PET/CT 7 d after UUO operation (UUO and contralateral, *n* = 6; healthy, *n* = 5). (B) Kidney volumes of UUO and healthy rabbits based on CT imaging (*n* = 3). (C) Picrosirius red staining shows dilation of renal tubules and tubulointerstitial fibrosis of renal cortex in UUO kidneys, whereas histologic findings are normal in contralateral kidney from same animal and in healthy control rabbits. Anti–CLEVER-1 immunohistochemical staining shows specific immunoreactivity in tissue macrophages in UUO kidney. Light brown unspecific background staining from urine flow can be observed in area of contralateral and healthy kidney tissue but not in UUO tissue. Scale bar is 200 μm.

The renal perfusion parameters in rabbits 7 d after the UUO operation are presented in Figure 2A and Supplemental Table 1. The ^15^O-radiowater PET analysis revealed that renal perfusion was significantly lower in the UUO renal cortex (2.00 ± 0.95 mL_blood_/mL_tissue_/min) than in the contralateral (5.57 ± 1.96 mL_blood_/mL_tissue_/min, *P* = 0.001) or healthy (5.25 ± 0.55 mL_blood_/mL_tissue_/min, *P* < 0.001) renal cortex.

On the basis of in vivo CT results, the volume of UUO-operated kidneys was enlarged, whereas the contralateral kidneys were similar in size to the kidneys from healthy rabbits (Fig. 2B).

The histopathologic analysis showed dilation of renal tubules, focal injury of tubular epithelial cells, and varying levels of inflammation in UUO kidneys. Additionally, picrosirius red staining showed tubulointerstitial fibrosis of the renal cortex in UUO kidneys. There were no histologic abnormalities in the contralateral kidneys from UUO rabbits or kidneys from healthy control rabbits (Fig. 2C). Anti–CLEVER-1 staining of UUO kidneys showed specific immunoreactivity on recruited macrophages in the tubulointerstitium. The contralateral kidneys or the kidneys from healthy rabbits did not show this pattern of immunoreactivity (Fig. 2C). No significant differences in staining were observed between the 7- and 14-d samples.

Anti–CLEVER-1 staining of spleen and kidney sections, as well as hematoxylin–eosin and picrosirius red staining of kidney sections obtained 7 d and 14 d after surgery, are presented in Supplemental Figures 2 and 3.

### Preparation of ^89^Zr-DFO-Bexmarilimab and Immunoreactivity

The DFO-NCS conjugation and labeling method enabled us to obtain a final formulation of ^89^Zr-DFO-bexmarilimab (Supplemental Fig. 4A) that was of excellent quality and without antibody aggregates or fragments, as measured by SDS-PAGE. The retained immunoreactivity of DFO-bexmarilimab was 87.5% ± 2.2% (*n* = 4), indicating that the modification with a chelator did not substantially alter antibody binding to CLEVER-1. Using 4 radiolabeled batches (*n* = 4), we determined that ^89^Zr-DFO-bexmarilimab had a radiochemical yield of 78.2% ± 4.2%, specific radioactivity of 76.1 ± 5.1 MBq/mg, and radioactivity concentration of 14.9 ± 4.4 MBq/mL. The radiochemical purity was 99.1% ± 0.3% when measured by ultrafiltration and 100% when measured by SDS-PAGE autoradiography (Supplemental Fig. 4B). When measured in a single batch, the immunoreactivity of ^89^Zr-DFO-bexmarilimab after radioactive decay (∼4 wk after radiolabeling, stored at −20°C) was similar to that of DFO-bexmarilimab (Supplemental Fig. 4C).

### ^89^Zr-DFO-Bexmarilimab PET/CT Imaging and Biodistribution

**FIGURE 3.**
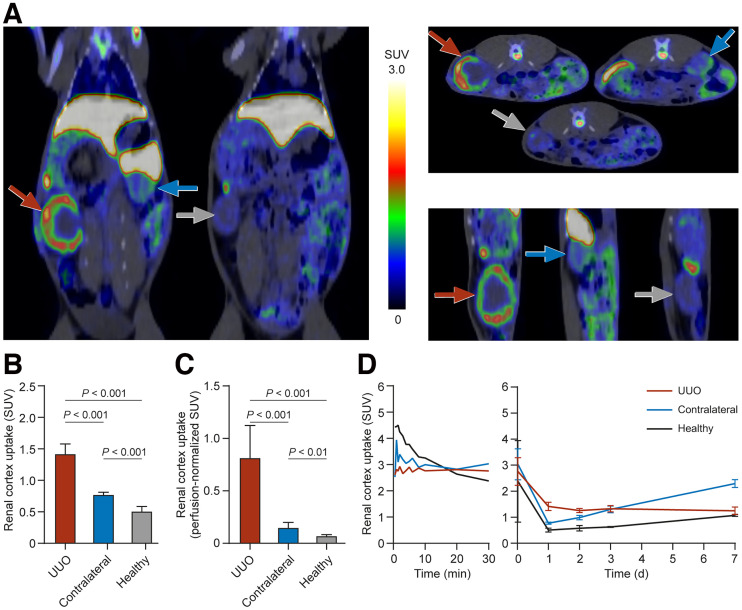
(A) Representative PET/CT images of 2 rabbits 24 h after single intravenous injection of ^89^Zr-DFO-bexmarilimab. Red arrows denote UUO kidneys; blue arrows, contralateral kidneys; and gray arrows, healthy kidneys. (B and C) Radioactivity concentration in renal cortex without perfusion correction (B) and with ^15^O-radiowater–based perfusion correction (C). (D) Time–activity curves for ^89^Zr-DFO-bexmarilimab uptake in renal cortex.

In UUO and healthy rabbits, in vivo PET/CT clearly visualized the liver and spleen and showed that there was some uptake of ^89^Zr-DFO-bexmarilimab in bone, bone marrow, and intestines (Fig. 3A). The highest radioactivity concentration after intravenous injection of ^89^Zr-DFO-bexmarilimab was in the liver, but the concentration decreased over time (Supplemental Figs. 5 and 6). The radioactivity concentration was always higher in the renal cortex than in the medulla, and the UUO kidney cortex was clearly visualized (Fig. 3A).

Uptake of ^89^Zr-DFO-bexmarilimab at 24 h after injection was significantly higher in the UUO renal cortex than in the contralateral or healthy renal cortex and was even more pronounced when normalized to the level of renal perfusion (Figs. 3B and 3C). Decay-corrected time–activity curves revealed that uptake of ^89^Zr-DFO-bexmarilimab in the UUO renal cortex remained constant after 24 h but increased over time in the contralateral renal cortex (Fig. 3D).

Supplemental Table 2 shows the ex vivo biodistribution of ^89^Zr-DFO-bexmarilimab in rabbits at 24 h and 7 d after injection. The organs with the highest radioactivity concentration were the liver, spleen, and bone or bone marrow; this result confirmed the in vivo PET/CT findings. Uptake was lowest in the brain.

### In Vivo Stability and Plasma Pharmacokinetics of ^89^Zr-DFO-Bexmarilimab

**FIGURE 4.**
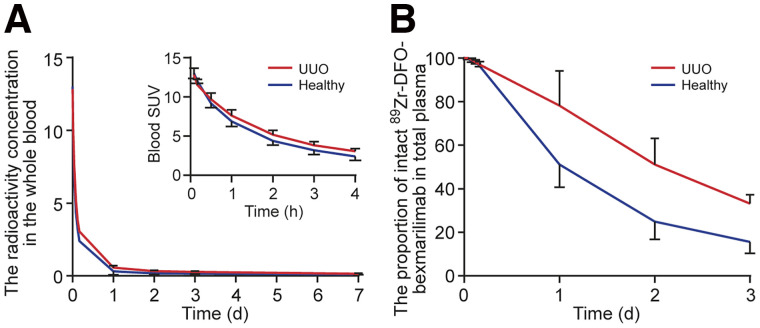
Distribution of ^89^Zr-DFO-bexmarilimab in rabbit blood circulation. (A) Radioactivity concentration in whole blood. (B) Proportion of intact ^89^Zr-DFO-bexmarilimab in total plasma. Lines represent mean values and bars are SDs of experiments.

The SDS-PAGE analysis of serial plasma samples from UUO rabbits showed that the proportion of intact ^89^Zr-DFO-bexmarilimab decreased from 97.0% ± 1.2% of total plasma radioactivity at 4 h after tracer injection to 78.2% ± 13.1%, 51.2% ± 9.8%, and 33.2% ± 3.4% at 24 h, 2 d, and 3 d, respectively. In healthy rabbits, the proportion of intact ^89^Zr-DFO-bexmarilimab was 96.2% ± 0.1%, 51.1% ± 8.5%, 24.9% ± 6.6%, and 15.6% ± 4.3% at 4 h, 24 h, 2 d, and 3 d, respectively, after tracer injection (Figs. 4A and 4B). Representative autoradiographs of SDS-PAGE are shown in Supplemental Figure 7.

The blood-to-plasma ratio of radioactivity was about 0.6 and did not change appreciably over the 7-d PET study (Supplemental Fig. 8). The plasma pharmacokinetic parameters are summarized in Supplemental Table 3. ^89^Zr-DFO-bexmarilimab had a relatively fast clearance from the blood circulation, with total clearance of 10.4 ± 2.1 mL/h (*n* = 3) in UUO rabbits and 17 ± 2.1 mL/h (*n* = 3) in healthy rabbits (*P* = 0.030).

### Digital Autoradiography

**FIGURE 5.**
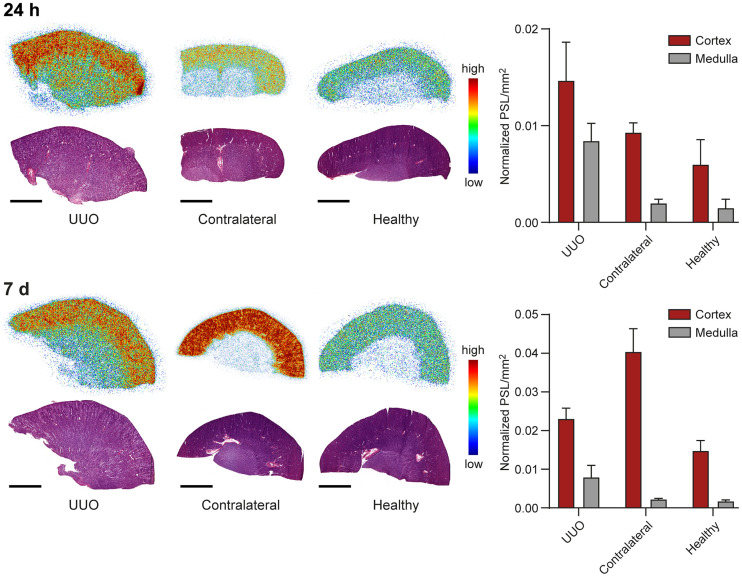
Representative ex vivo digital autoradiographs and hematoxylin–eosin staining of 20-μm kidney cryosections and quantification data 24 h and 7 d after ^89^Zr-DFO-bexmarilimab injection. Scale bar is 5 mm.

Digital autoradiography of rabbit kidney cryosections combined with hematoxylin–eosin staining confirmed that ^89^Zr-DFO-bexmarilimab was retained in the renal cortex (Fig. 5). Despite impaired renal perfusion in the UUO kidney, uptake 24 h after injection was higher in the UUO renal cortex and medulla than in the contralateral and healthy kidneys (*P* < 0.05). At 7 d after injection, uptake was highest in the contralateral renal cortex; this finding is consistent with the results of PET/CT studies and ex vivo γ-counting.

### Radiation Dose Estimates

The human residence times (the normalized numbers of disintegrations) for the various source organs and the remainder of the body extrapolated from healthy rabbit data are listed in Supplemental Table 4. Supplemental Figure 9 shows the time–activity curves of the organs on which the dosimetry calculation using OLINDA/EXM 2.1 was based. The estimates of the organ doses given in Supplemental Table 5 were calculated for a 70-kg man. The organs with the highest doses were the liver (5.860 ± 1.100 mGy/MBq), gallbladder wall (2.580 ± 0.420 mGy/MBq), and adrenal glands (1.777 ± 0.240 mGy/MBq). The mean effective dose (ICRP 103 (14)) was calculated as 0.702 ± 0.051 mSv/MBq. For example, a 37-MBq dose of ^89^Zr-DFO-bexmarilimab would likely result in an effective dose of 26 mSv.

## DISCUSSION

Bexmarilimab is a new humanized anti–CLEVER-1 antibody currently in clinical immunotherapy trials. In addition to its potential use as an immunotherapeutic, we are interested in determining whether the antibody is suitable for immuno-PET imaging. The information presented from this study increases our understanding of its pharmacokinetics and in vivo biodistribution and provides estimates for the human radiation dose, which will support future clinical translation of ^89^Zr-DFO-bexmarilimab immuno-PET.

To enable ^89^Zr-radiolabeling, bexmarilimab was successfully conjugated with a DFO chelator without compromising immunoreactivity, that is, 87.5% of the immunoreactivity was retained. Subsequent radiolabeling resulted in the formation of ^89^Zr-DFO-bexmarilimab that had a high radiochemical yield and high radiochemical purity. SDS-PAGE was a particularly suitable method to evaluate purity because it enabled all radioactive substances with different molecular sizes, whether free ^89^Zr or antibody aggregates, to be separated from the intact tracer in the same assay. Most importantly, conjugating bexmarilimab with ^89^Zr and DFO did not lead to loss of immunoreactivity. This finding is important, since ^89^Zr-DFO-bexmarilimab is intended for use in clinical PET applications.

CLEVER-1, as observed by anti–CLEVER-1 immunohistochemistry, was clearly expressed on macrophages residing in the UUO kidney, which were not present in the contralateral and healthy kidneys. PET/CT imaging, ex vivo γ-counting, and autoradiography analysis similarly showed significant differences in the uptake of ^89^Zr-DFO-bexmarilimab into UUO kidneys compared with the contralateral and healthy kidneys at 24 h after injection. ^89^Zr-DFO-bexmarilimab showed a surprisingly fast clearance coupled with fast initial uptake in the target tissues.

Although the kidneys show uptake of ^89^Zr-DFO-bexmarilimab at 24 h after injection, they are also a likely route of excretion of radiometabolites and fragments. We hypothesize that the increased uptake of tracer in the contralateral and healthy kidneys 7 d after injection, as shown by PET/CT and ex vivo results, is due to uptake of radiometabolites, given that the level of radiometabolites in the bloodstream increases in the days after the injection of ^89^Zr-DFO-bexmarilimab. In UUO and healthy rabbits, radiometabolites consisted of, on average, 84% and 67%, respectively, of the total plasma radioactivity on day 3 after injection. Almost no intact ^89^Zr-DFO-bexmarilimab was detected on day 7, although the total plasma radioactivity at that time was too low for accurate detection. An approximate doubling of radioactivity uptake in the contralateral kidney compared with the healthy kidney on day 7 would support this hypothesis, as the contralateral kidney compensates for the loss of UUO kidney function. A likely source of circulating metabolites may be the liver, as a decrease in liver radioactivity was observed after the 24- and 48-h time points (Supplemental Fig. 6).

The fast uptake and clearance of ^89^Zr-DFO-bexmarilimab highlight the importance of determining the optimal time window for PET studies in achieving meaningful results. In this study, the optimal time window was clearly at 24 h, when tracer accumulation in the UUO kidney had stabilized, blood radioactivity had diminished, and radiometabolites had not yet begun to accumulate in the healthy and contralateral kidneys.

In general, the uptake of ^89^Zr-DFO-bexmarilimab in the liver and spleen corresponded to previously reported CLEVER-1 expression in human tissues (3–5). However, although we confirmed CLEVER-1 expression by immunohistochemical staining in the spleen and UUO kidneys, we did not analyze the liver and other tissues that possibly express CLEVER-1.

The estimated human radiation burden due to a single intravenous ^89^Zr-DFO-bexmarilimab injection is comparable to that of other ^89^Zr-labeled monoclonal antibodies (15–17) and is suitable for clinical studies. In this study, scaling between rabbit and human data was performed using organ and whole-body masses of rabbits and humans. Despite similarities between species, the accuracy of extrapolation of biokinetic data from laboratory animals to humans is uncertain, particularly for the liver because of qualitative differences between species in the handling of many elements by this organ.

## CONCLUSION

On the basis of the preclinical results of this study, including the estimated human radiation burden, ^89^Zr-DFO-bexmarilimab is suitable for future clinical PET studies.

## DISCLOSURE

Maija Hollmén and Sirpa Jalkanen own stock in Faron Pharmaceuticals. The study was financially supported by grants from Business Finland, the Jane and Aatos Erkko Foundation, the Academy of Finland (#350117), and the Finnish Cultural Foundation. Olli Moisio is a PhD student partially supported by the Drug Research Doctoral Program of the University of Turku Graduate School and the doctoral module of the InFLAMES Flagship. No other potential conflict of interest relevant to this article was reported.

## Notes

KEY POINTS **QUESTION:** Are the characteristics of CLEVER-1–targeted ^89^Zr-DFO-bexmarilimab suitable for prospective human PET studies? **PERTINENT FINDINGS:** This preclinical rabbit study revealed that intravenously administered ^89^Zr-DFO-bexmarilimab was able to detect fibrosis associated with abundant CLEVER-1–positive cells. The estimated human effective dose was within the safe limits for potential human use. **IMPLICATIONS FOR PATIENT CARE:** Clinical studies to determine whether ^89^Zr-DFO-bexmarilimab-PET is suitable for, for example, stratifying patients for bexmarilimab treatment, evaluating treatment efficacy, and monitoring disease progression, may be justified.
